# Effects of low-frequency noise from wind turbines on heart rate variability in healthy individuals

**DOI:** 10.1038/s41598-021-97107-8

**Published:** 2021-09-08

**Authors:** Chun-Hsiang Chiu, Shih-Chun Candice Lung, Nathan Chen, Jing-Shiang Hwang, Ming-Chien Mark Tsou

**Affiliations:** 1 Research Center for Environmental Changes, Academia Sinica, Taipei, Taiwan; 2 Department of Atmospheric Sciences, National Taiwan University, Taipei, Taiwan; 3 Institute of Environmental Health, National Taiwan University, Taipei, Taiwan; 4 Institute of Statistical Science, Academia Sinica, Taipei, Taiwan

**Keywords:** Environmental sciences, Risk factors

## Abstract

Wind turbines generate low-frequency noise (LFN, 20–200 Hz), which poses health risks to nearby residents. This study aimed to assess heart rate variability (HRV) responses to LFN exposure and to evaluate the LFN exposure (dB, *L*_Aeq_) inside households located near wind turbines. Thirty subjects living within a 500 m radius of wind turbines were recruited. The field campaigns for LFN (*L*_Aeq_) and HRV monitoring were carried out in July and December 2018. A generalized additive mixed model was employed to evaluate the relationship between HRV changes and LFN. The results suggested that the standard deviations of all the normal to normal R–R intervals were reduced significantly, by 3.39%, with a 95% CI = (0.15%, 6.52%) per 7.86 dB (*L*_Aeq_) of LFN in the exposure range of 38.2–57.1 dB (*L*_Aeq_). The indoor LFN exposure (*L*_Aeq_) ranged between 30.7 and 43.4 dB (*L*_Aeq_) at a distance of 124–330 m from wind turbines. Moreover, households built with concrete and equipped with airtight windows showed the highest LFN difference of 13.7 dB between indoors and outdoors. In view of the adverse health impacts of LFN exposure, there should be regulations on the requisite distances of wind turbines from residential communities for health protection.

## Introduction

Wind energy is used around the world as a source of clean energy. However, wind turbines generate low-frequency noise (LFN) in the range of 20–200 Hz^1–4^. As many community complaints have centered around the LFN from wind turbines^5^, it is important to evaluate the health impacts of LFN on residents near wind farms.

LFN exposure has been found to cause a variety of health conditions. Exposure to LFN from wind turbines results in headaches, difficulty concentrating, irritability, fatigue, dizziness, tinnitus, aural pain sleep disturbances, and annoyance^6–19^. Clinically, exposure to LFN from wind turbines may cause increased risk of epilepsy, cardiovascular effects, and coronary artery disease^20,21^. It was also found that exposure to noise (including LFN) may have an impact on heart rate variability (HRV)^22,23^. HRV is the variation over time of the period between adjacent heartbeats^24^, which is an indicator of the activities of the autonomic nervous system, consisting of the sympathetic nervous system (SNS) and parasympathetic nervous system (PNS). Autonomic imbalance usually represents a hyperactive SNS and a hypoactive PNS and results in reduced HRV. An autonomic imbalance may increase the morbidity and mortality of cardiovascular diseases^25^. A review paper indicated that road traffic noise may overactivate the hypothalamic-pituitary-adrenocortical axis (HPA) and sympathetic-adrenal-medullar axis (SAM), increase the blood pressure and reduce HRV, and finally affect the cardiovascular system^26^. A recent study analyzing 658 measurements of HRV obtained from 10 healthy males (18–40 years old) indicated reductions in HRV due to environmental LFN exposure^27^. However, few studies have specifically examined the effect of LFN from wind turbines on HRV in healthy individuals; thus, this was the aim of this study.

In view of the adverse health impacts of noise exposure, many countries and international organizations have established regulations for noise control. These regulations are set for noise in the full spectrum of human hearing (20–20 k Hz). The Ministry of Environment of Finland set limits for wind farm noise of 45 dB (*L*_Aeq_) during the day and 40 dB (*L*_Aeq_) during the night^28^. In the United Kingdom, the fixed limit for turbine noise is 40 dB (*L*_Aeq_) for the daytime and 43 dB (*L*_Aeq_) for the nighttime^29^. In the United States, noise levels of ≤ 55 dB (*L*_Aeq_) are set for outdoors in residential areas, farms, and other outdoor areas as requisites for public health protection, and levels of 45 dB are set for indoor residential areas, hospitals, and schools^30,31^. In addition to the full noise spectrum, the Taiwan Environmental Protection Administration (EPA) also established regulations for LFN to avoid impacts on residents, since wind farms have been set up very close to residential communities. The LFN standards for wind turbines in the daytime (7 a.m.–7 p.m.) and evening (7 p.m.–10 p.m.) are 39 dB (*L*_Aeq_) for environments requiring tranquility such as residential areas, 44 dB (*L*_Aeq_) for mixed residential and commercial/industrial areas, and 47 dB (*L*_Aeq_) for industrial areas; those at nighttime (10 p.m.–7 a.m.) are 36, 36, 41, and 44 dB (*L*_Aeq_), respectively^32^. This study assessed the LFN in the indoor environments of households near wind turbines to evaluate whether the LFN levels meet the Taiwan EPA standards.

One of the most important factors influencing residential noise exposure from wind turbines is the distance of the wind turbine from the observer^33^. For example, at a distance of 120–500 m, the measured turbine noise levels decreased by 3–5 dB (*L*_Aeq_), while at a distance of 1000 m the noise was reduced by 6–7 dB (*L*_Aeq_)^34^. Hansen et al. reported variations in indoor LFN levels (15–45 dB (*L*_Aeq_)) for two households (houses made of sandstone/concrete/iron or bricks with windows remaining closed or half open) at different distances from wind turbines^35^. This study assessed the indoor/outdoor differences in LFN exposure in several households located at varying distances from wind turbines. Our main focus was on the indoor LFN levels in several recruited households; we did not intend to conduct a comprehensive evaluation of the influential factors. These households serve the purpose of demonstrating the potential impacts of influential factors.

Besides distance from turbines, building materials also affect indoor LFN exposure. This work assessed the indoor LFN levels for several recruited households with different building materials and open/closed windows to illustrate their potential impacts. It is known that materials have different sound absorption coefficients^36^. The overall sound pressure level and spectrum of external noise change when transmitted to the interior of a building^37^. Mid- and high-frequency noises are selectively attenuated by roofs and walls, causing the building structure to function like an LFN pass filter^38^. Outdoor to indoor noise reduction generally decreases with frequency, which is related to housing construction and room dimensions^35^. Factors contributing to indoor/outdoor noise reduction also include structural resonances, room modes, and coupling between the air volume inside the residence and the stiffness of the walls, roofs, and ceilings^35^. It is known that the appropriate choice of construction materials and designs can contribute to LFN exposure reduction for residents. Hence, these factors are not evaluated comprehensively in this study.

Taiwan is a small and highly populated island. Wind farms have been set up near residential communities, affecting the day-to-day lives of the residents. The hypothesis of this study is that LFN from wind turbines might affect HRV of residents. In order to verify the hypothesis of this study, we defined two objectives: to evaluate the LFN and HRV relationship with an intervention design and to assesses the actual LFN exposure of the community residents. This investigation is the first in Asia examining the impact of LFN from wind turbines on the HRV of healthy residents. In addition, the variations in LFN exposure inside several residences constructed of different building materials are examined. The findings of this study would serve as a useful reference for Asian countries planning to launch or promote wind power generation.

## Materials and methods

The following sub-sections describe the study design, monitoring equipment, LFN and HRV monitoring strategies, household LFN exposure monitoring, and data analysis.

### Study design

There were two types of field monitors used in this work. The first one was used to assess the changes in HRV of the recruited subjects while taking LFN measurements at the same time at two designated sites with an intervention design; this is called “LFN and HRV monitoring”. The second one was used to assess the LFN levels in the indoor and outdoor environments of several households; we intended to use it to assess whether the daily LFN exposures of residents were at the same range as those obtained in the first intervention monitoring.

### Environmental monitoring devices

The sound level meter NL-62 (Rion, Japan), which complies with the IEC 61672-1, 2002 Class 1 and ISO 7196: 1995 applicable standards, was used for measuring the LFN. Measurements were analyzed using the frequency analysis program NX-62RT, with the choice of 1/3 of an octave and at intervals of 100 ms. Its band central frequencies were 20, 25, 31.5, 40, 50, 63, 80, 100, 125, 160, and 200 Hz. In addition, windscreens WS-10 and WS-15 were used in indoor and outdoor measurements, respectively, and equipped with rainproof features^39^ to reduce the wind noise. The average acoustical intensity (dB, *L*_Aeq_) was measured over a 5 min period. Moreover, the wind speed in the outdoor environment was measured by a wind speed smart sensor (S-WSB-M003), while the temperature and relative humidity (RH) in both indoor and outdoor environments were obtained using HOBO U12 data loggers (Onset Computer Corporation, Bourne, MA). Meteorological data were collected at a 5 min resolution.

### HRV monitoring device

RootiRx (RootiCare^®^, Rooti Labs Ltd., Taipei, Taiwan), a wearable electrocardiogram (ECG) recorder, was employed to provide data on heart rate, HRV indicators, and motion in three dimensions (https://www.rooticare.com). RootiRx has received medical-device certifications from the EU, the US, and Taiwan. This compact device (62 × 22.5 × 8.45 mm, 14 ± 1 g) is attached to an electrode sticker (32.7 × 115 × 5.2 mm, 3.2 g) and can operate under a temperature of 0–40 °C and an RH of 20–93%. RootiRx has been evaluated against a standard 12-lead Holter monitor in 33 healthy subjects for 24 h^40^. The overall average beat per minute correlation between BeyondCare® (another name for RootiRx) and the standard 12-lead Holter was found to be 0.98. The mean percentage of invalid measurements was 1.6% for RootiRx and 1.7% for Holter. These findings indicate that the overall performance of RootiRx and Holter is similar. Moreover, the same study used RootiRx to assess cardiac arrhythmias in 67 subjects. The mean analyzable wear time was found to be 93.6%, and its water-resistant design enabled 73.5% of the participants to take a shower^40^. It was used in our earlier work to assess impacts on HRV from air pollution^41^. We applied RootiRx for subjects recruited in a community close to wind turbines.

All the ECG signals collected from RootiRx at the sampling rate of 500 Hz were downloaded and analyzed using previously validated proprietary algorithms provided by Rooti Labs Limited^40^. After automatically producing the data of HRV indices and excluding the artifacts (non-normal beats signals) by the proprietary algorithms, experienced research scientists in Rooti Labs Limited reviewed each batch of data. Overall, the sampling frequency (> 200 Hz) and duration of recording for investigating short-term HRV (5 min) met the recording requirements in the guidelines of the Task Force of the European Society of Cardiology and the North American Society of Pacing and Electrophysiology^42^, and the quality of RootiRx was compared to that of the standard Holter monitor as mentioned above^40^.

### LFN and HRV monitoring

The targeted community was located within a 500 m radius of wind turbines in western Taiwan. Public recruitment meetings were held to introduce our study objectives and plan. The inclusion criteria were residents aged between 20 and 80 years and non-smokers. The exclusion criteria were hearing impairments, high blood pressure, known heart disease, history of heart attack and heart surgery, irregular heart rhythm, and medications taken that would affect HRV. Thirty subjects were recruited with written informed consent. Then, face-to-face interviews were conducted to obtain detailed demographic data as well as information on the building materials of the residences, the types of windows installed, and the residents’ habits of keeping windows open or closed (√: fully open; x: slightly open or fully closed). This study was designed and conducted complying with the guidelines of the standards set by the latest revision of the Declaration of Helsinki of the World Medical Association and was reviewed and approved by the Institutional Review Board of Academia Sinica (IRB No. AS-IRB01-18025). All participants in this study provided signed informed consent.

**Figure 1:**
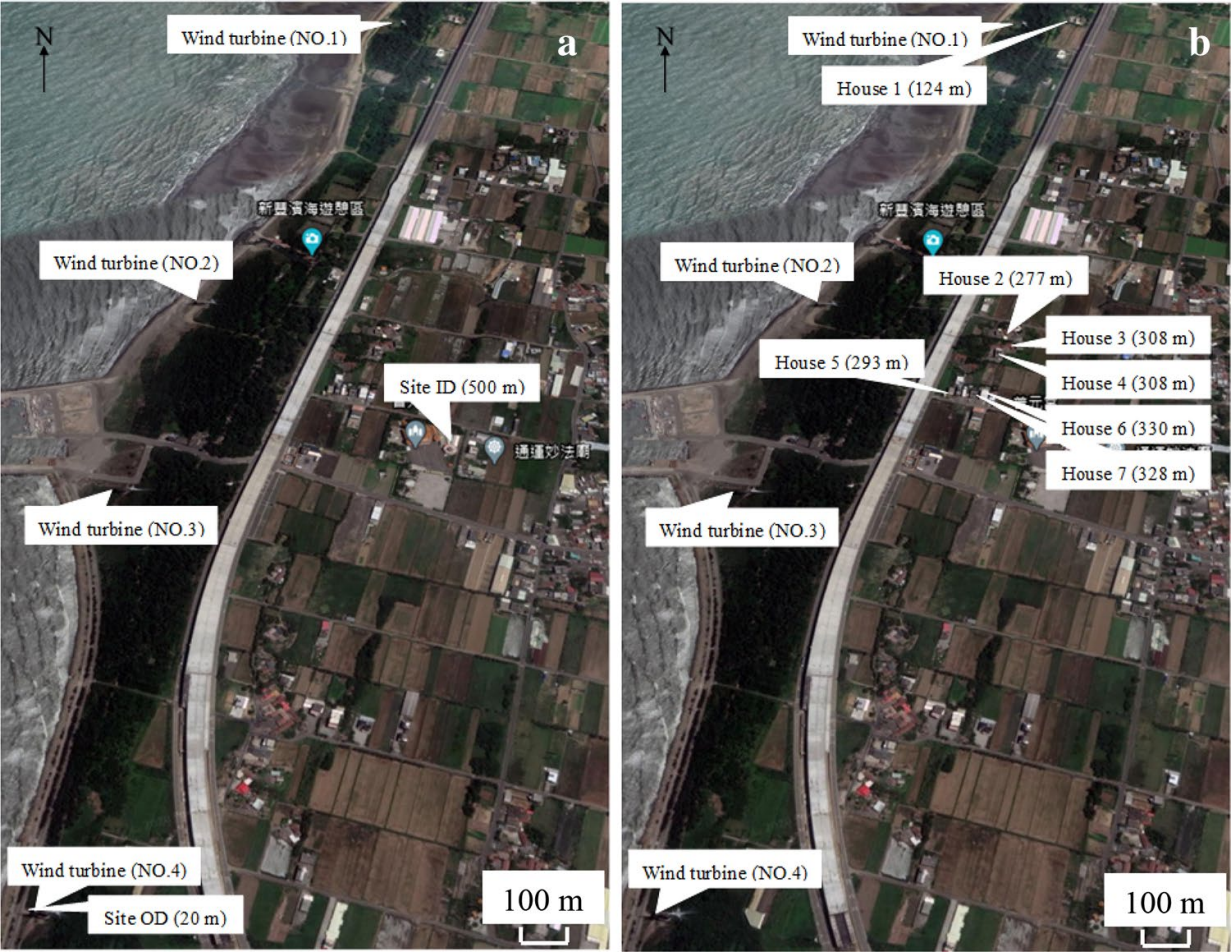
Schematics showing the locations of **(a)** field monitoring sites and **(b)** household monitoring residences relative to wind turbines^69^.

The field campaigns for LFN and HRV monitoring were carried out in July (summer) and December (winter) 2018. In each month, the field campaign lasted for two days on the first weekend of the month and was repeated again on the third weekend. Two sites were selected: an outdoor site (Site OD) and an indoor site (Site ID). Figure 1a illustrates the locations of two sites relative to the wind turbines. The distances were measured from the nearest wind turbine. Site OD was located in close proximity to the wind turbines 20 m away, where people would be exposed to relatively high levels of LFN. In contrast, Site ID was located at a distance of 500 m from the wind turbines; thus, people would be exposed to relatively low levels of LFN. The two sites were about a 5 min drive apart. The recruited subjects were gathered on the two sites to acquire different levels of LFN exposure, as described below. This intervention design aimed to make sure the subjects were exposed to LFN from wind turbines during our monitoring periods in a similar manner to residents who live near wind turbines in their daily lives are.

On Day 1, RootiRx was patched onto the chest of each subject at Site ID. Then, all the subjects were instructed to fill out a time-activity diary (TAD) at 30 min intervals regarding their microenvironments, activities, and major exposure sources encountered, if any, during that 30 min period. The exposure sources listed included vehicles, electronics, wind turbines, machines, trains, aircrafts, music, conversations, and others (please specify).

On Day 2, all subjects gathered first at Site ID, the activity center of the study community. They were equally assigned into two groups, Group A and Group B, with similar age and gender distributions. Then, Group A was taken to Site OD. Transport was provided when shuttling between the two sites to avoid their HRV being affected by other factors. At both sites, the subjects were asked to sit in the chairs provided and the 1-h LFN monitoring commenced. The LFN levels were matched to the HRV of the subjects to assess the associations of HRV and LFN in a later data analysis. To prevent exposure to sunlight from affecting the subjects’ HRV, a tent was pitched for monitoring at Site OD. Subjects were engaged only in low-intensity activities during the monitoring. At Site OD, subjects had their heights, weights, and blood pressures measured in the tent, while at Site ID, they listened to a talk on different types of energy and their applications. These activities were not vigorous and would not cause mood swings, thus having a minimum impact on HRV. According to our observations, all subjects were sitting or performing low-intensity activities that had minimal impact on the HRV during the monitoring at Site OD and Site ID. We tried our best to maintain similar conditions for both groups. Nevertheless, environmental factors could differ, such as temperature, humidity, and wind speed. After an hour, the two groups swapped sites, with Group A taken to Site ID and Group B to Site OD, and another one-hour LFN monitoring was repeated. At the end of monitoring on Day 2, RootiRx was removed from each subject. For field campaigns conducted in December, warm drinks were provided at both sites to minimize the effect of cold weather on subjects’ HRV.

The environmental LFN levels were simultaneously measured at each site when the subjects wearing RootiRx gathered, as described above. In addition, environmental variables, including wind speed, temperature, and RH, were measured during field campaigns at both sites. At Site OD, instruments, including Rion NL-62, HOBO U12, and HOBO S-WSB-M003, were set up 3 m from the subjects about 1.7 m above the ground (roughly the height of human ears); meanwhile, at Site ID, instruments, including Rion NL-62 and HOBO U12, were set up on a table 1 m above the ground, approximately 2 m from the wall, and 3 m from the subjects.

It is noteworthy that regular LFN patterns (waves with an amplitude of 1–2 dB) occurred due to the running of the wind turbines. Additionally, the LFN from wind turbines is affected by wind speeds; the observed regular patterns moved gradually up or down, changing with wind speeds. If certain peaks occurred within these regular patterns, they were presumably from other sources (such as road vehicles and aircraft)^41^. We checked the raw data visually and manually and removed those sudden peaks. The percentage excluded was below 1%. After these quality control and quality assurance procedures, we were confident that the LFN in our dataset was from wind turbines rather than from other sources.

### Household LFN exposure monitoring

The purpose of the household indoor and outdoor monitoring was to assess whether the everyday LFN exposures of residents were in similar ranges to the LFN exposures measured in the aforementioned field monitoring, which purposely staged the subjects staying at site OD. To assess the actual LFN exposure of subjects in their everyday life, the continuous 48-h monitoring of indoor and outdoor LFN was conducted in households in August–September (summer) and December (winter). Among 30 subjects, only seven households agreed on household monitoring. The distances of these seven households from the nearest wind turbine are shown in Fig. 1b, from 124 (House 1) to 330 m (House 6). Three households were monitored in summer and six in winter, with two residences (Houses 3 and 5) monitored in both seasons. For outdoor monitoring, the Rion NL-62 was set up approximately 2 m from the outside walls of the studied households at 1.7 m above ground. For indoor monitoring, Rion NL-62 was set up on a table in the living room of the studied households about 2 m from the wall at 1 m above ground. In order to avoid testing bias in real world situations for indoor and outdoor monitoring that had different temperatures, humidity, and wind speeds, we tried our best to maintain similar conditions such as instrument parameter settings and monitoring height and time.

### Data analysis

Data cleaning procedures were carried out for the LFN and HRV data. The 5 min environmental variables (LFN (*L*_Aeq_), temperature, RH, and wind speed) were calculated by averaging the data collected each minute during field campaigns. Though driven by winds of varying speed, wind turbines rotate steadily, and the LFN thus generated should be regular and uniform. A sudden increase in the LFN level exceeding 10 dB (*L*_Aeq_) should be attributed to other sources (such as road vehicles and airplanes). Therefore, to ensure that the LFN data collected during field campaigns were from wind turbines, acute rises in the LFN of more than 10 dB (*L*_Aeq_) at Site OD were excluded from data analysis. This exclusion criterion was not applied to Site ID, where diverse sources of LFN, as described later, were present, resulting in wider variations and more irregularity in the LFN trends observed.

The HRV responses to LFN exposures were assessed using a time-domain parameter, namely, the standard deviation of all normal to normal R-R intervals (SDNNs); and a frequency-domain parameter, namely, the ratio of low-frequency (LF; 0.04–0.15 Hz) to high-frequency power (HF; 0.15–0.4 Hz) (LF/HF). Even though breathing rate may affect the short-term time-domain measurements, LF and HF^24^, previous studies nonetheless found that short-term exposure to PM_2.5_ was significantly associated with the decreases in SDNN and/or increases in LF/HF^41,43–45^. In addition, subjects were asked to only engage in low-intensity activities in the field campaigns, and they had to complete the time-activity diaries to record their activities. Moreover, the activity intensity of subjects was considered in the models to control the subjects’ breathing rate. Since SDNN is the most commonly used HRV index and LF/HF reflects the sympatho-vagal balance, we selected SDNN and LF/HF as the indicators in this study. Abnormal data with SDNNs greater than 250 and an LF/HF smaller than 0.1 were excluded from the analysis. HRV data in 5 min intervals with a missing rate greater than 20% were not included in further analysis^40^. After data cleaning, the average analyzable wear time of RootiRx in this work was 86.2%. Signal loss was attributed to the poor contact of the RootiRx patch worn by subjects in action. The data for LFN and HRV indicators were matched in 5 min intervals for exposure-health evaluation. Studies indicated that the 5 min HRV, as compared to a 24-h measurement, was a strong indicator of cardiac events in the normal population and patients^46–49^. Long-term (24 h) measurements for HRV reflect the overall change in the heart rate under nonspecific, changing conditions, and short-term measurements offer more practical advantages, including easy application in a clinical setting and a simplified data process^49,50^.

The associations between LFN and log_10_-transformed HRV indicators (SDNN and LF/HF) in 5 min intervals were analyzed using the general additive mixed model (GAMM, R Version 3.5.0). The model was adjusted for wind speed, temperature, age, gender (female was coded as 0 and male as 1), body mass index (BMI, body weight/(height^2^) < 24 was coded as 0 and ≥ 24 as 1), activity intensity (mG: milli-gravitational constant, 6.674 × 10^−14^ m^3^/kg s^2^), and the interaction term of age and activity intensity. Activity intensity and temperature were adjusted using thin-plate spline in the GAMM models for log_10_(SDNN) and log_10_(LF/HF), respectively, as these variables were statistically significant in that model. Subjects were treated as random effect intercepts to adjust for individual differences. The cut-off for BMI was 24, following the guidelines of the Ministry of Health and Welfare, Taiwan, which takes BMI ≥ 24 as overweight^51^. Moreover, in view of the strong correlation between temperature and RH, RH was excluded from the model. The effect estimate (*β*) of LFN was transformed into the percentage change of HRV indicator per interquartile range (IQR) of the corresponding covariate, which can be presented as $\left[{10}^{\left(\beta \times \mathrm{IQR}\right)}-1\right]\times 100\%$. The corresponding 95% confidence interval (CI) was presented as $[{10}^{\left(\beta \mp 1.96\times SE\right)\times {\text{IQR}}}-1]\times 100\%$, where *SE* is the standard error of the *β* estimate. Note that three asterisks (***), two asterisks (**) and one asterisks (*) showed *p* < 0.001, 0.001 < *p* < 0.05, and 0.05 < *p* < 0.1, respectively in the statistical analysis.

For household monitoring, indoor and outdoor LFN (dB, *L*_Aeq_) measurements obtained from the 48 h monitoring were divided into three different time periods, namely, daytime (7 a.m. to 7 p.m.), evening (7 p.m. to 10 p.m.), and nighttime (10 p.m. to 7 a.m.), for comparison against the LFN standards designated by the Taiwan EPA. Moreover, the impacts of building materials, types of windows, and habits of keeping windows open or closed on the LFN indoor/outdoor differences were explored. In view of the insignificant effect of slightly open windows on LFN transmission^52^, slightly open and fully closed windows were grouped into the same category in contrast to fully open windows.

## Results

### Distribution of demographics, LFN and HRV data

Among the 29 subjects recruited, 11 males and 13 females participated in the summer field campaign, while 9 males and 10 females participated in the winter field campaign, with 14 subjects (8 males, 6 females) participating in both seasons. Among the 29 subjects recruited in our study, LFN and HRV monitoring was carried out for 2 h (at Site OD and ID). In addition, the environmental and LFN measurements were in 5 min resolution. The ideal sample sizes of LFN and HRV monitoring were 1460. However, abnormal data with SDNNs greater than 250, an LF/HF smaller than 0.1, and HRV data in 5 min intervals with a missing rate greater than 20% were excluded from the analysis. After data cleaning, the actual sample size was 1259 (Table 1). Table 1 lists the demographic characteristics of the subjects. The age of the subjects ranged from 22 to 75 years. The average BMI of subjects was 23.9 ± 2.8 kg/m^2^ in summer and 24.3 ± 2.9 kg/m^2^ in winter. As shown in Table 1, for the summer and winter measurements, the mean SDNNs were 61.7 and 66.5 ms, while the mean LF/HFs were 1.9 and 1.1, respectively. The mean levels of LFN exposure were 43.5 ± 2.9 at Site OD and 50.4 ± 0.8 dB (*L*_Aeq_) at Site ID in summer and 49.1 ± 6.9 and 42.3 ± 1.8 dB (*L*_Aeq_), respectively, in winter. According to previous studies, these three factors are important factors for HRV^41,44,53^. The distributions of age, gender, and BMI in summer and winter are shown in Supplementary Table S1. Although these distribution factors were not significantly different between summer and winter, these factors were still included in the following GAMM analysis.

**Table 1:** Demographic characteristics of study subjects and environmental and LFN measurements with a 5 min resolution (n = 1259).

Variable	Summer (n = 574)			Winter (n = 685)		
	Mean ± SD	Min	Max	Mean ± SD	Min	Max
Age (years)	54 ± 14	22	75	59 ± 14	33	75
BMI (kg/m^2^)	23.9 ± 2.8	19.0	30.3	24.3 ± 2.9	21.1	34.0
Temperature (°C)	35.8 ± 3.9	31.5	41.0	22.6 ± 1.5	19.6	24.7
Relative humidity (%)	55.6 ± 10.6	40.8	67.2	73.5 ± 3.4	66.9	80.8
Wind speed (m/s)	0.95 ± 0.78	0	2.01	0.72 ± 0.79	0	2.42
Activity intensity (mG)	1891 ± 398	1201	3546	1781 ± 337	1173	3323
SDNN (ms)	61.7 ± 29.4	8.9	167.3	66.5 ± 29.2	11.7	180.9
SDNN at site OD (ms)	65.3 ± 30.8	8.9	167.3	68.8 ± 31.4	11.7	180.9
SDNN at site ID (ms)	55.8 ± 26.1	11.4	144.5	64.2 ± 26.5	12.8	146.7
LF/HF	1.9 ± 2.2	0.08	19.1	1.1 ± 1.2	0.09	13.3
LF/HF at site OD	1.7 ± 2.1	0.1	19.1	1.1 ± 1.3	0.1	13.3
LF/HF at site ID	2.1 ± 2.4	0.1	14.2	1.2 ± 1.1	0.1	8.5
LFN (*L*_Aeq,_ dB)	46.1 ± 4.1	38.3	53.5	45.8 ± 6.1	38.2	57.1
LFN at site OD (*L*_Aeq,_ dB)	43.5 ± 2.9	38.3	53.5	49.1 ± 6.9	40.5	57.1
LFN at site ID (*L*_Aeq,_ dB)	50.4 ± 0.8	48.6	52.5	42.3 ± 1.8	38.2	45.5

Site OD: 20 m from wind turbines; Site ID: 500 m from wind turbines.

*BMI* body mass index, *mG* milli-gravitational constant, 6.674 × 10^−14^ m^3^/kg s^2^, *SDNN* standard deviation of normal to normal R–R interval in 5 min resolution, *LF/HF* low frequency to high frequency ratio in 5 min resolution,* LFN* levels of the entire monitoring period.

### Impacts of LFN exposure on HRV

One of the main objectives of this study was to evaluate the potential impacts of LFN on HRV in terms of SDNN and LF/HF. Table 2 shows the 5 min percentage changes of HRV indicators per interquartile range (IQR) increase in LFN. GAMM analysis yielded 7.86 dB (*L*_Aeq_) as the IQR of LFN. After adjusting for confounding factors, the SDNN was reduced by 3.39% (95% CI: 0.15–6.5%) per 7.86 dB (*L*_Aeq_) of LFN with a statistical significance of *p* < 0.05. In other words, with an increase of 1 dB (*L*_Aeq_) in LFN, the SDNN decreased by 0.43%. In contrast, the reduction in LH/HF per IQR increase in LFN was much smaller and not statistically significant. These results revealed a significant association between LFN exposure and changes in HRV, especially in SDNN, indicating the potential health impacts of exposure to LFN.

**Table 2:** Estimated percentage changes (95% CI) in (a) the 5 min SDNN and (b) the 5 min LF/HF per interquartile range (IQR) (7.86 *L*_Aeq,_ dB) increase in LFN (n = 1259).

	Coefficient estimates	
	5 min SDNN	5 min LF/HF
LFN^a^	−3.39** (−6.52 to −0.15)	−0.37 (−6.44 to 6.10)
Wind speed	12.7*** (7.80 to 17.9)	-9.07* (-17.6 to 0.37)
Temperature	−0.025 (−4.20 to 4.34)	–^d^
Age	−6.43 (−23.1 to 13.8)	−31.5** (−48.4 to −9.11)
Gender^b^	−5.02 (−25.5 to 21.07)	58.7** (11.8 to 125.4)
BMI^c^	2.17 (–8.80 to 14.4)	−2.73 (−17.5 to 14.6)
Activity intensity	–^d^	−8.29*** (−12.6 to −3.81)

***: *p* < 0.001; **: 0.001 < *p* < 0.05; *: 0.05 < *p* < 0.1.

^a^LFN was treated as a continuous variable.

^b^Gender: female was coded as 0 and male as 1.

^c^BMI < 24 was coded as 0 and BMI ≥ 24 as 1.

^d^Temperature and activity intensity were adjusted using thin-plate spline for SDNN and LF/HF GAMM models, respectively.

### LFN exposure in residential households

In order to assess whether the subjects’ daily LFN exposure was in a similar range to our “LFN and HRV monitoring”, we conducted household LFN exposure monitoring for seven recruited households. The households’ average LFN levels were 34.8 $\pm$ 6.9 dB and 43.4 $\pm$ 5.7 dB for indoors and outdoors, respectively. As shown in Table 3a, the indoor LFN exposure during the 24-h period among different households ranged from 30.7 to 43.4 dB (*L*_Aeq_). Moreover, the maximum indoor LFN exposure of residents in their households in the daytime ranged between 39.7 and 56.7 dB (*L*_Aeq_), which was similar to the range recorded at Site ID (38.2 and 52.5 dB). As shown in Table 3b, the outdoor LFN measured during a 24-h period among different households ranged from 38.2 to 50.0 dB (*L*_Aeq_) in summer and 38.9 to 44.6 dB (*L*_Aeq_) in winter. These household data were slightly lower than the field results at Site OD (38.3–53.5 dB, *L*_Aeq_, in summer and 40.5–57.1 dB, *L*_Aeq_, in winter), which was located much closer (20 m) to the turbines than the households. As shown in Table 3c, the CN residences (Houses 1, 2, 6, and 7) had larger indoor–outdoor differences (range, 6.6–11.2 dB (*L*_Aeq_)) than the concrete with brick (CB) residence (House 3; (range, 5.8–8.5 dB (*L*_Aeq_)). The results indicated that CN had a higher LFN insulation compared with CB.

**Table 3:** *L*_Aeq, 5-min_ LFN (dB, *L*_Aeq_) measured at different households (a) indoors, (b) outdoors, and (c) outdoors minus indoors.

Seasons	Households	*L*_Aeq, 5-min_ indoor (dB)											Building material	^a^Distance (m)	^b^Habits of opening windows
		24-h period	n	Daytime (7 am–7 pm)	Max	n	Evening (7 pm–10 pm)	Max	n	Nighttime (10 pm–7 am)	Max	n			
**(a) Indoor**															
Summer	House 1	43.4 ± 3.1	472	44.2 ± 2.5^c^	49.6^c^	284	45.0 ± 4.1^c^	50.0^c^	64	40.8 ± 2.3^c^	48.5^c^	124	CN	124	x
	House 3	32.4 ± 5.1	364	33.8 ± 4.4	54.1^c^	270	32.6 ± 4.4	45.8^c^	25	26.5 ± 3.6	33.4	69	CB	308	x
	House 5	30.7 ± 3.0	404	32.1 ± 2.4	39.7^c^	253	28.6 ± 1.8	33.7^c^	36	28.2 ± 2.5	36.2^c^	115	CA	293	√
Winter	House 2	33.8 ± 8.1	513	39.4 ± 4.7^c^	51.8^c^	225	38.2 ± 4.9	48.3^c^	72	26.4 ± 5.4	47.9^c^	216	CN	277	x
	House 3	33.7 ± 8.3	813	34.4 ± 8.1	55.6^c^	385	34.7 ± 7.3	56.5^c^	104	32.5 ± 8.7	48.1^c^	324	CB	308	x
	House 4	37.9 ± 2.1	580	38.2 ± 2.8	52.2^c^	291	37.6 ± 0.6	40.0^c^	72	37.7 ± 0.8^c^	46.3^c^	216	CN	308	√
	House 5	30.7 ± 4.5	587	32.5 ± 3.8	49.0^c^	299	30.8 ± 2.9	39.7^c^	72	28.1 ± 4.5	42.2^c^	216	CA	293	x
	House 6	32.9 ± 6.3	600	36.4 ± 5.0	56.7^c^	312	33.7 ± 6.4	52.2^c^	72	27.6 ± 3.9	42.7^c^	216	CN	330	x
	House 7	37.7 ± 5.9	589	40.4 ± 5.3^c^	53.5^c^	302	41.1 ± 5.1^c^	53.8^c^	71	32.8 ± 3.1	44.3^c^	216	CN	328	x
**(b) Outdoor**															
Summer	House 1	50.0 ± 5.2	472	49.5 ± 5.0	55.3	284	50.2 ± 1.8	53.8	64	51.1 ± 6.5	56.0	124	CN	124	x
	House 3	38.2 ± 3.9	364	39.3 ± 2.8	47.8	270	38.0 ± 3.5	45.2	25	33.8 ± 4.6	45.6	69	CB	308	x
	House 5	44.4 ± 3.7	404	46.0 ± 2.9	54.5	253	41.8 ± 3.3	51.3	36	41.7 ± 3.5	51.5	115	CA	293	√
Winter	House 2	42.2 ± 6.1	513	46.4 ± 4.4	63.9	225	43.1 ± 3.4	54.7	72	37.5 ± 4.9	51.5	216	CN	277	x
	House 3	38.9 ± 4.8	273	41.2 ± 5.5	60.8	129	39.0 ± 3.7	47.8	36	36.2 ± 2.3	42.0	108	CB	308	x
	House 4	40.9 ± 5.6	595	44.2 ± 4.5	63.0	299	41.7 ± 3.8	61.7	80	36.0 ± 3.8	50.9	216	CN	308	√
	House 5	44.3 ± 4.5	587	46.8 ± 3.2	59.1	299	43.2 ± 3.4	52.6	72	41.2 ± 4.1	53.5	216	CA	293	x
	House 6	43.8 ± 4.1	585	45.8 ± 3.8	63.9	297	43.8 ± 4.3	64.1	72	41.1 ± 2.7	50.7	216	CN	330	x
	House 7	44.6 ± 4.0	589	46.8 ± 3.3	61.0	302	44.5 ± 3.5	58.9	71	41.7 ± 3.1	52.1	216	CN	328	x

Seasons	Households	*L*_Aeq, 5-min_ indoor (dB)								Building material	^b^Habits of opening windows				
		24-h period	n	Daytime (7 am–7 pm)	n	Evening (7 pm–10 pm)	n	Nighttime (10 pm–7 am)	n						
**(c) Indoor–outdoor**															
Summer	House 1	6.6 ± 5.4	472	5.4 ± 4.6	284	5.2 ± 3.3	64	10.3 ± 6.2	124	CN	x				
	House 3	5.8 ± 4.7	364	5.5 ± 4.7	270	5.4 ± 5.6	25	7.2 ± 3.8	69	CB	x				
	House 5	13.7 ± 2.0	404	13.9 ± 1.9	253	13.2 ± 2.7	36	13.5 ± 2.1	115	CA	√				
Winter	House 2	8.4 ± 6.7	513	6.9 ± 6.6	225	4.9 ± 6.3	72	11.1 ± 6.0	216	CN	x				
	House 3	8.5 ± 4.1	273	7.8 ± 4.6	129	9.1 ± 3.6	36	9.0 ± 3.3	108	CB	x				
	House 4	2.8 ± 5.0	579	5.8 ± 4.7	292	3.6 ± 2.7	72	1.7 ± 3.8	216	CN	√				
	House 5	13.7 ± 3.9	586	14.4 ± 2.7	298	12.3 ± 3.3	72	13.1 ± 5.2	216	CA	x				
	House 6	11.2 ± 3.9	585	9.8 ± 3.5	297	10.1 ± 5.5	72	13.5 ± 2.5	216	CN	x				
	House 7	6.9 ± 4.1	589	6.4 ± 4.7	302	3.4 ± 4.2	71	8.8 ± 1.3	216	CN	x				

*CN* concrete, *CB* concrete + brick, *CA* concrete + airtight windows.

^a^Distance from the nearest wind turbine (m).

^b^Habits of opening windows: √: fully open; x: slightly open or fully closed.

^c^LFN exposure levels exceeding the respective standards designated by the Taiwan EPA.

## Discussion

According to the results of distribution of demographics and LFN and HRV data, while the winter data (Site OD > Site ID) support our hypothesis that close proximity to wind turbines results in higher LFN exposure, the summer findings (Site ID > Site OD) of a higher LFN recorded with the wind turbines distant away imply a greater contribution to LFN from other sources indoors. Reviewing the differences between the sites in the two seasons showed that the use of fans indoor in hot weather was a potential source of LFN at Site ID. Summer in Taiwan is hot, and Site ID was equipped with fans for ventilation. Another possible source was human conversation. The voice of a typical adult male has a fundamental frequency from 85 to 180 Hz and that of a typical adult female has a fundamental frequency from 165 to 255 Hz^54^.

To evaluate the contribution of these two potential sources, a follow-up assessment of the LFN at Site ID was performed. First, 15 min LFN measurements were taken for indoor background noise first without any fan or conversation, then they were taken with one turned-on household fan (medium-sized) roughly 3 m away from the NL-62, and finally they were taken with the ongoing conversation of five persons (three males and two females) at 2–5 m away from the NL-62. The LFN at a 1 min resolution was assessed. The results show that the average indoor background LFN was 32.5 ± 1.4 dB (*L*_Aeq_) without fan use and conversation, 32.5 ± 1.4 dB (*L*_Aeq_) with the fan turned on, and 44.1 ± 2.2 dB (*L*_Aeq_) with ongoing conversation. As can be seen, with the fan turned on, the indoor LFN recorded was the same as the background LFN. In contrast, with ongoing conversation, the indoor LFN recorded was 11.6 dB (*L*_Aeq_) higher than the background LFN. In other words, the summer findings (Site ID > Site OD) of the LFN recorded indoors surpassing that of the LFN recorded outdoors with wind turbines nearby can be attributed to the ongoing conversation indoors at the activity center.

Recio et al. (2016) indicated that exposure to repetitive noise may reduce emotional overload, but at the cost of increasing the allostatic load^26^. Therefore, the risk of adverse health outcomes may increase due to serious physiological alterations. In addition to decreasing the HRV, repetitive exposure to noise may induce the systemic inflammation and oxidative stress due to SAM and HPA overactivation. Moreover, systemic inflammation and oxidative stress may also cause autonomic imbalance^55^, which affects HRV. The findings of the impacts of LFN exposure on HRV in our study are consistent with previous observations of reduced HRV due to LFN exposure in the US^27^. In a study on 10 healthy males, SDNN was reduced by 16% (95% CI: 6.1–26%) during 40 min LFN exposure as compared with no noise exposure. As stated above, few studies have assessed the impacts of LFN on HRV. With the increasing emphasis on renewable energy, a growing trend of more turbines being built for wind power can be expected. Hence, it is both timely and necessary to conduct more assessments on the potential health impacts of LFN generated by wind turbines.

In addition to LFN, studies have also indicated the possible effect of environmental noise on HRV. For 110 German adults exposed to daytime noise, the SDNN reduced by 0.67% for a 5 dBA increase when *L*_eq_ $\ge$ 65 dBA^56^. Another study also showed a decrease from 17.42 to 17.7 ms for a 10 dBA increase above the background noise (45 dBA) in forty college-going male volunteers exposed to traffic noise^57^; the SDNN reduction was roughly 0.2% per 1 dBA. Additionally, an SDNN reduction was observed for another important environment factor, PM_2.5_. A significant decrease in SDNN (0.51%; 95% CI: 0.01–1.01%) was associated with a 10 µg/m^3^ increase in PM_2.5_ for Japanese patients aged 20–90 years^58^. A meta-analysis of 33 panel studies in North America, Asia, and Europe showed that a 0.92% reduction in SDNN was observed for a 10 μg/m^3^ increase in short-term PM_2.5_ exposure^59^. Wang et al. showed that 0.39% (95% CI: − 0.72%, − 0.06%) and 0.92% (95% CI: − 2.14%, 0.31%) reductions were observed for short-term and long-term exposures to a 10 μg/m^3^ increase in PM_2.5_, respectively, for adults aged above 55 years old^60^. In addition to this, SDNN was decreased from 54.7 to 39.6% with an increase in ambient temperatures from 17 to 38 °C (reduced by 0.72% per 1 °C) in 28 healthy young subjects^61^. In comparison, we found that SDNN reduction (0.43% per 1 dB) due to LFN from wind turbines was slightly higher than the reduction in traffic noise exposure > 65 dBA (0.13% per 1 dB), in a similar range or slightly lower than those with a 10 μg/m^3^ increase in PM_2.5_ exposure (0.39 to 0.92%) and slightly lower than those with ambient temperatures (0.72% per 1 °C). In short, the impact of LFN from wind turbines on HRV was higher than that from traffic noise and lower than that from PM_2.5_ and ambient temperature.

The results of the impacts of LFN exposure on HRV also imply the absence or minimal lag effect of LFN on SDNN. During field monitoring, the subjects were shuttled between Site OD (high LFN exposure) and Site ID (low LFN exposure). Should there be a lag effect, their differences in LFN would be small, and the impact of LFN on SDNN would be insignificant, resulting in similar SDNNs at both sites. Nevertheless, the field monitoring results indicated a significant reduction in SDNN at Site ID (55.8 ms in summer and 64.2 ms in winter) compared to those at Site OD (65.3 ms in summer and 68.8 ms in winter). Hence, there was either no or a negligible lag effect. This observation was consistent with the results of a study conducted in Germany that showed that during routine activities, a 5 dB increase in *L*_Aeq_ ≥ 65 dB (20–20 k Hz) was not associated with lagged SDNN change^56^.

Besides LFN, wind speed is the only environmental variable with a significant impact on SDNN and LF/HF, but with opposing trends of changes (Table 2). In this study, wind speed and temperature were environmental variables adjusted in the GAMM of LFN on SDNN and LH/HF. The exact mechanism by which these variables impact the two HRV indicators remains to be explored.

The SDNN decreased with increasing age but did not reach statistical significance in our work. In the literature, studies have found a significant association of age with SDNN. For example, Kim and Woo found that SDNN significantly decreased with increasing age in 2748 males and 735 females in Korea^62^. Voss et al. also indicated an inverse association of age with SDNN for 1906 Germans aged 25–74 years^63^. Furthermore, population studies in the Netherlands found that SDNN decreased continuously from birth to old age for 28,827 participants with ages ranging from 11 days to 91 years^64^. In addition, as seen in Table 2, female subjects had a higher SDNN than male subjects, again not reaching statistical significance. Previous studies have also indicated that female subjects had a significantly higher SDNN than male subjects^62,64^. Our study results showed a trend of increased association of SDNN and age and higher SDNN in females, consistent with the results of previous studies. Nevertheless, the sample size in this work may be the reason for the statistical insignificance of these results.

One of the concerns of this study is the sample size. We have to emphasize that the objective of this study was to evaluate relationships between the LFN and HRV, which may be affected by many factors. There were some procedures for controlling these factors and increasing the sample size. First, the subjects had to complete the questionnaires and time-activity dairies to collect the information about these factors. Next, we had designed the conditions for indoor and outdoor environments, and we also controlled the subjects’ activities in the field to control the impacts of environmental and activity factors on HRV. We also used the repeated measure design to increase the sample size. There were some challenges in field recruitment such as the limited number of residents in the studied community. In addition, the duration and funding of the study and the compliance of residents may also have affected the sample size. According to the above reasons, it was difficult to estimate the sample size before recruitment. Therefore, we tried to recruit as many subjects as possible in the field and tried to control the confounding factors for HRV. Moreover, we used the GAMM to assess the relationships between the LFN and HRV. The GAMM can control the subject variability with the random effect. We tried to increase the statistical power using the above procedures to determine whether the relationships between the LFN and HRV were significant. Based on our results, an increase in LFN was associated with a decrease in HRV.

Compared to the results of LFN exposure in residential households, previous studies have reported wind turbine LFN of 15–45 dB indoors in residences in Australia situated 870–3100 m from wind turbines^35^, and indoor LFN levels of 0–10 dB during wind turbine operational periods for residences situated 1500 m from wind turbines in Australia^65^. In comparison, the maximum indoor LFN exposure during a 24-h period (43.4 dB) in our monitoring was similar to that reported (45 dB) by Hansen et al.^35^ and higher than that (10 dB) reported by Evans et al.^65^.

Our results also confirmed that these residents indeed were exposed to similar LFN levels as in the field campaign at the site OD. Therefore, HRV impacts from the LFN exposure evaluated in the field campaigns could be found in the daily lives of these residents. Hansen et al. and Evans et al. also reported that the LFN exposure levels from wind turbines for outdoor measurements were 25–40 dB and 21–25 dB, respectively^35,65^. Our monitoring of outdoor LFN exposure (38.2–50.0 dB) was higher than their results. The seven households in this study were located closer (124–330 m) to the turbines than the households in Hansen et al. (870–3100 m)^35^ and Evans et al. (1500 m) were^65^.

Table 3a also shows that the indoor LFN exposure levels (dB*, L*_Aeq_) in most households were higher in the daytime than in the evening and nighttime. The higher LFN exposure level in the daytime could be attributed to other sources of background noise in addition to turbine-generated noise, while the LFN at nighttime presumably came mainly from wind turbines. House 1 recorded the highest mean and maximum nighttime LFN exposure (40.8 and 48.5 dB (*L*_Aeq_), respectively), attributed to its close proximity to the wind turbines. According to the Guidelines for Community Noise^66^, for a good night’s sleep the equivalent sound level should not exceed 30 dB (*L*_Aeq_) for continuous background noise. However, the average indoor LFN levels at night in Houses 1, 3, 4, and 7 were above 30 dB (*L*_Aeq_), and 100% of the 5 min nighttime observations recorded in Houses 1 and 4 exceeded 30 dB (*L*_Aeq_), implying that turbine-generated LFN may affect residents' quality of sleep at night in these households.

Taiwan’s EPA designates different noise standards in residential areas for different times of day: daytime (39 dB, *L*_Aeq_, 7 am to 7 pm), evening (39 dB, *L*_Aeq_, 7 pm to 10 pm), and nighttime (36 dB, *L*_Aeq_, 10 pm to 7 am)^32^. According to these guidelines, House 1 (daytime, evening, and nighttime), House 2 (daytime), House 4 (nighttime), and House 7 (daytime and evening) had LFN exposure levels exceeding the respective standards designated by the Taiwan EPA (Table 3a). Among these residences monitored, residents at House 1 had higher LFN exposures from wind turbines round the clock, indicating that they exceeded the LFN standards of Taiwan’s EPA 99.6%, 89.1%, and 96.8% of the time for the daytime, evening, and nighttime, respectively. The impacts of distance, building materials, and having windows open/closed on the LFN can be illustrated by our cases, as briefly discussed below. The significant influence of distance from turbines on the indoor LFN exposure level is best illustrated by House 1. With the shortest distance from wind turbines, House 1 had the highest mean LFN exposure, both indoors and outdoors, during the 24-h period, with its indoor LFN exposure ranging from 40.8 to 45.0 dB (*L*_Aeq_) and the maximum level reaching 50 dB (*L*_Aeq_) in the evening. However, House 6, the farthest (330 m) among the seven households monitored, did not record the lowest mean LFN exposure. Instead, the lowest LFN exposure of 30.7 dB (*L*_Aeq_) in both seasons was recorded inside House 5 (concrete with airtight windows (CA)), the only residence with airtight windows installed. Windows serve as sound attenuation^67^. Although the resident of House 5 indicated a habit of keeping windows fully open in the summer (Table 3), the windows were actually closed during the monitoring period according to our observation. Moreover, House 5 had only one resident, and the windows were kept closed when the house was empty. A single-member household with less ongoing conversation would imply the absence or negligible contribution of this indoor LFN source (unlike the situation at Site ID). Moreover, keeping airtight windows closed most of the time contributes to low sound transmission. The soundproofing effectiveness of airtight windows is evidenced by the largest indoor–outdoor difference in LFN of 13.7 dB (*L*_Aeq_) recorded for House 5 in both seasons (Table 3c).

Houses 3 and 4 were located at equal distances (308 m) from the nearest turbine, but their average indoor LFN exposure differed by 4 dB (33.7 dB (*L*_Aeq_) and 37.9 dB (*L*_Aeq_), respectively (Table 3a). The results indicated that though it had been built with concrete (CN), House 4 had a higher indoor LFN recorded, which can be attributed to the fully open windows, resulting in poorer sound insulation compared with House 3 with its fully closed windows. Therefore, the impacts of opening the windows on the indoor LFN were more significant than those of building materials in this case.

The impacts of building materials on LFN were not a focus of our study. However, the indoor–outdoor LFN difference in seven households presented certain indications of their impacts. In summary, distance from turbines, building materials used, types of windows installed, and whether they are open or closed all had impacts on the indoor LFN levels in our study.

### Recommendations and study limitations

The present results show the adverse impact of LFN exposure on HRV. For public health protection, there should be regulations on the requisite distances of wind turbines from residential communities. In Taiwan, wind farms are owned by large corporations, and distance regulations would prevent these operators from reaping benefits at the expense of nearby residents suffering from long-term LFN disturbance and adverse health impacts. In order to reduce LFN transport from outdoors to indoors, we recommend that the windows should be kept closed, especially at nighttime because LFN is most noticeable at night. In addition, airtight windows are good for sound insulation. Therefore, we recommend that residences in close proximity to wind turbines should be equipped with airtight windows. As far as we know, a small number of wind turbine firms provided certain funding to install the airtight windows for residents living nearby. We suggest that this funding should be required by governmental agencies.

This study has some limitations. Firstly, at Site ID for the households monitored, indoor LFN was caused by wind turbines, but there may also exist other indoor LFN sources, such as indoor ventilation devices, ongoing conversations, or television, which have not been thoroughly explored. Secondly, our sample size was small due to difficulties in recruitment. Nevertheless, the findings obtained from the 30 subjects still demonstrated the impacts of LFN on HRV changes. Thirdly, the average analyzable wear time (86.2%) was lower than that (93.6%) found in previous research^41^. Nevertheless, data loss occurred randomly and did not undermine the validity of the present findings. Fourthly, except for LFN sources, the psychological stress from other sources was not assessed in this study, which may affect HRV. However, if the psychological stress from other sources and LFN did not always concurrently affect the HRV, the psychological stress from other sources would not affect the coefficients of LFN. Finally, air pollutants such as PM_2.5_ levels were not measured in this study. Exposure to PM_2.5_ may cause a reduced HRV. Previous study found that evaluated PM_2.5_ exposure and noise exposure were both associated with changes in HRV^68^. However, they only measured the levels of noise rather than the frequency of noise. The interaction of LFN with PM_2.5_ should be evaluated in future studies.

## Conclusion

LFN from wind turbines is potentially annoying to residents living nearby and affects human health. This study assessed the response of HRV indicators (SDNN and LF/HF) to LFN exposure and evaluated the LFN exposure inside households located near wind turbines. The results showed the association of changes in HRV with LFN exposure and an SDNN reduction of 0.43% with an increase of 1 dB (*L*_Aeq_) in LFN. The households’ average LFN levels were 34.8 $\pm$ 6.9 and 43.4 $\pm$ 5.7 dB for indoors and outdoors, respectively. In addition, the average indoor LFN levels at nighttime in four of the seven households monitored were above 30 dB (*L*_Aeq_), the threshold for good sleep quality. Taiwan has a high population density, and wind farms have been set up near residential communities. In view of the adverse health impacts of exposure to turbine-generated LFN, it is recommended that the government set regulations on the requisite distances of wind turbines from residences, for houses near wind turbines to be equipped with airtight windows for sound insulation, and for residents living in close proximity to wind turbines to have their windows closed most of the time to reduce LFN transmission.

## Supplementary Information

Supplementary Table S1.
