# Supplementary material for: Effects of low-frequency noise from wind turbines on heart rate variability in healthy individuals
Source: Sci Rep. 2021 Sep 8;11:17817. doi: 10.1038/s41598-021-97107-8 (PMC8426498; doi:10.1038/s41598-021-97107-8)
Supplement: Supplementary file 1 — Supplementary Table S1. [file 41598_2021_97107_MOESM1_ESM.docx]

**Supplemental Material**

Table S1. Distribution of age, gender and BMI in summer and winter.

|  | Summer (n=24) | Winter (n=19) |  |
| --- | --- | --- | --- |
| Variables | n (%) | n (%) | p-value |
| Age |  |  |  |
| 20 to 49 | 11 (45.8) | 7 (36.8) | 0.553 |
| 50 to 75 | 13 (51.2) | 12 (63.2) |  |
| Gender |  |  |  |
| Male | 11 (45.8) | 9 (47.4) | 0.920 |
| Female | 13 (54.2) | 10 (52.6) |  |
| BMI ^a^ (kg/m^2^) |  |  |  |
| < 24 | 13 (54.2) | 13 (68.4) | 0.342 |
| ≥ 24 | 11 (45.8) | 6 (31.6) |  |

a: BMI: Body Mass Index.
